# A computational approach for designing D-proteins with non-canonical amino acid optimised binding affinity

**DOI:** 10.1371/journal.pone.0187524

**Published:** 2017-11-06

**Authors:** Michael Garton, Maryam Sayadi, Philip M. Kim

**Affiliations:** 1 Donnelly Centre for Cellular and Biomolecular Research, University of Toronto, Toronto, Canada; 2 Department of Molecular Genetics, University of Toronto, Toronto, Canada; 3 Department of Computer Science, University of Toronto, Toronto, Canada; Universidade Nova de Lisboa Instituto de Tecnologia Quimica e Biologica, PORTUGAL

## Abstract

Redesigning protein surface topology to improve target binding holds great promise in the search for highly selective therapeutics. While significant binding improvements can be achieved using natural amino acids, the introduction of non-canonical residues vastly increases sequence space and thus the chance to significantly out-compete native partners. The potency of protein inhibitors can be further enhanced by synthesising mirror image, D-amino versions. This renders them non-immunogenic and makes them highly resistant to proteolytic degradation. Current experimental design methods often preclude the use of D-amino acids and non-canonical amino acids for a variety of reasons. To address this, we build an *in silico* pipeline for D-protein designs featuring non-canonical amino acids. For a test scaffold we use an existing D-protein inhibitor of VEGF: D-RFX001. We benchmark the approach by recapitulating previous experimental optimisation with canonical amino acids. Subsequent incorporation of non-canonical amino acids allows designs that are predicted to improve binding affinity by up to -7.18 kcal/mol.

## Introduction

Engineering of biomolecules to alter or augment their function continues to broaden in scope for both design novelty and the range of applications. Recent advances in medicine[1], agriculture[2], carbon capture[3], biosensors[4], and advanced materials[5] are the direct results of success in protein design. The vast majority of such designs have been constructed using the twenty canonical amino acids (CAAs) found in nature. However, much greater versatility is available by introducing non-canonical amino acids (NCAAs) to the repertoire. The number of NCAAs now available vastly extends the potential for design of new proteins with novel functions, modified binding specificity, and improved affinities. To date there are more than 200 commercially available NCAAs and the list is growing[6]. These range from simple methylated phenylalanines to more exotic functional group substitutions and unusually large hydrophobic constructs[7]. This diversity allows more nuanced design of surface shape, core packing, and hotspot interaction geometry. However, the almost infinite increase in sequence space does pose difficulties for experimental design due to the constraints imposed by current library sizes[8] and the greatly elevated cost of NCAAs compared to CAAs. For these reasons, computational protein design is an attractive approach for the design of proteins comprising NCAAs.

Another aspect of protein design that can make a computational approach prudent or necessary is when D-amino acids are incorporated. Almost all amino acids have chirality and therefore exist in either dextrorotary (D) or levorotary (L) forms–so-called because of their influence on plane-polarised light. D-amino acids are occasionally found in nature, such as in venoms, antibiotics, and peptidoglycan cell walls, but they are extremely rare[9,10]. Nature is peculiarly homo-chiral and the L-enantiomer prevails. D-protein non-immunogenic and degradation resistance properties stem from this phenomenon as D-proteins are not recognised by their L-protein interaction partners (such as proteases). As proteolytic degradation is a major barrier to deploying proteins as pharmacological agents[11], D-protein properties confer significant advantages for biomedicine design. The D-protein properties of being both non-immunogenic and resistant to proteolytic degradation make them ideal for biomedical applications. It allows better cell penetration[12,13] as well as increased gut, blood plasma, and intra-cellular half-life[14]. Together this can impart potency improvements (compared to L-counterparts) of up to five orders of magnitude[15].

Engineering D-amino therapeutic peptides can be achieved experimentally using mirror image phage display (MIPD). Targets are synthesised in D-space and used as bait for a randomised L-amino peptide library[16]. Successful candidate peptides/proteins subsequently made with D-amino acids bind the native L-protein target with the same affinity as their reverse. Experiments are currently limited to a target D-protein size of ~150 residues by commercial synthesis techniques, although synthesis of up to 312 residues have been reported[17]. This size limitation largely precludes membrane proteins, which comprise ~60% of all drug targets. Using a computational approach can circumvent the size limitation and allow access to any target that has–or can be–structurally solved or modelled. Other issues that can make experimental design difficult or impossible are the requirements for chaperones or obligate hetero-dimeric partners. L-chaperones are unlikely to specifically recognise their D-protein substrate because the topology is very different. Folding is therefore usually precluded[18] (although an exception has been demonstrated for DapA folding by GroEL/ES[17]–thought to proceed using nonspecific hydrophobic interactions). Computational design represents a viable alternative where such experimental design issues persist.

Here we describe a computational approach for designing D-proteins with NCAA optimised binding affinity. A series of computational tools juxtaposed with manual curation provides a quick and inexpensive way to generate a tractable set of designs. The approach utilises Rosetta design, visual inspection, and thermodynamic integration (TI) in a systematic elimination process. TI is currently amongst the most accurate computational techniques for free energy calculation–consistently shown to match experimental energy values with minimal error[19–22]. Drug–target[20], protein–protein[23], protein–DNA[24], and protein–peptide[25] interaction energies have all been accurately measured using TI. The approach described here utilises TI to screen Rosetta generated design candidates and thus enhance prediction accuracy.

For a test model we use D-RFX001[26], a published D-protein inhibitor of the vascular endothelial growth factor (VEGF). VEGF is an important target for arresting pathologic angiogenesis, most notably a feature of carcinogenesis, but also of macular degeneration (AMD) [27]. AMD is the most common cause of blindness in adults over fifty-five[28]. Ocular injectable therapeutics are available but the drug half life is relatively short[29]. Protease resistant D-proteins with NCAA optimised binding affinity may facilitate substantial improvements in half-life and potency. D-RFX001 was originally engineered by mirror synthesis and optimised with CAAs using phage libraries[26]. The CAA optimisation allowed preliminary validation of the *in silico* method, on which basis NCAAs were used to further optimise binding affinity. Our approach predicts that incorporation of specific NCAAs at key positions will increase D-RFX001 affinity for VEGF. This approach may prove useful in light of the growing demand for more exotic molecules and the experimental constraints associated with them.

## Methods

### Model preparation and MD simulation

PDB structure 4GLN containing the VEGF homodimer in complex with engineered protein D-RFX001 was retrieved from the PDB. Water molecules and other ions and ligands were removed as well as one of the two D-peptide ligands removed (to minimise computation cost). The remaining structure was prepared for Amber MD simulation using the WHATIF web interface[30] to build in any missing atoms. Correct protonation states were identified and annotated. The model was subsequently energy minimised using a combination of steepest descent and conjugate gradient methods. Following this it was equilibrated and heated over 100 ps to 300K and positional restraints were gradually removed. Restraints were completely removed and full equilibration was achieved after a further 12 ns of MD. Root mean square deviation (RMSD) was calculated to determine convergence. Following equilibration, a 50 ns trajectory was produced for the analysis. Simulations were carried out in explicit solvent comprising a 12 nm3 box of TIP3P water using TLEAP in AMBER 12[31]. Sodium counter-ions were added for overall charge neutrality and periodic boundary conditions were applied. Bonds to hydrogen were constrained using SHAKE[32] and a 2 fs time step was used. The particle mesh Ewald[33] algorithm was used to treat long-range electrostatic interactions and the non-bonded cut-off was set at 12.0 Å. MD calculations were carried out with the SANDER module of AMBER 12 in conjunction with the FF99SB Stony Brook forcefield[34]. A Berendsen thermostat and barostat was used throughout for both temperature and pressure regulation[35]. During calculations a snapshot was saved every 2 ps. Clustering of the of the 50 ns production trajectory was carried out using the MMTSB toolset[36] (kclust tool) to produce a manageably sized, representative ensemble for design calculations. Radius was set to 2.0 Å and maxerr to 1. This generated a set of 14 backbones representative of the space sampled during the simulation. These, together with the original crystal structure, were used as the models for mutation calculations using Rosetta.

### Rosetta calculations

Rosetta modelling calculations were carried out using the Python based interface: PyRosetta[37]. The native VEGF-D-RFX001 complex PDB file was first reformatted for compatibility with Rosetta, followed by calculation of the native Rosetta energy score using the ‘full atom’ scoring function. This calculation was repeated 100 times to obtain an average energy score and standard deviation. Each interface side chain of D-RFX001 was then separately mutated to each of the 20 CAAs and the energy score recalculated. Again each variant was repeated 100 times to obtain averaged scores. For both native and all of the mutant variants, energy score calculations were preceded by full repacking of all side-chains using the ‘packmover2’ function. This searches for the lowest energy configuration of rotamers using the Dunbrack library[38]. Sampling of rotamers is necessarily discretized and sampling from the Dunbrack library was set to include additional rotamers at two full standard deviations from the mean chi angle. Differences between native and mutant scores were calculated and negative energy change values were used to generate logos using WebLogo[39]. A PDB structure was also generated for each of the mutations to aid manual curation of the output. Calculation of mutations to NCAAs followed the same protocol as for CAAs. Parameters for NCAAs were not included in the Rosetta package, so a previously validated set of 114 parameterized NCAAs with backbone dependent rotamer libraries was obtained and used[40]. Example scripts and input files can be found in the supplementary material.

### Manual curation

Because TI is very computationally expensive, it is prudent to manually curate the Rosetta output before proceeding to this step. Mutations where negative change in the energy score does not make sense structurally and biochemically can be eliminated. Visual inspection of each such mutation was carried by loading the structures output by Rosetta into Chimera[41] and PyMol[42]. Hydrogen bonds, electrostatic interactions and relevant inter-chain atom-atom distances were displayed. These features–together with shape complementarity–were used to decide predicted beneficial mutation was valid. A common example of elimination was mutation to large hydrophobic residues that had no atoms <5Å from VEGF. Another issue was an apparent Rosetta scoring function bias toward GLY and ALA. Approximately 70% of the candidates from Rosetta were removed at this step.

### Thermodynamic integration calculations

**Fig 1:**
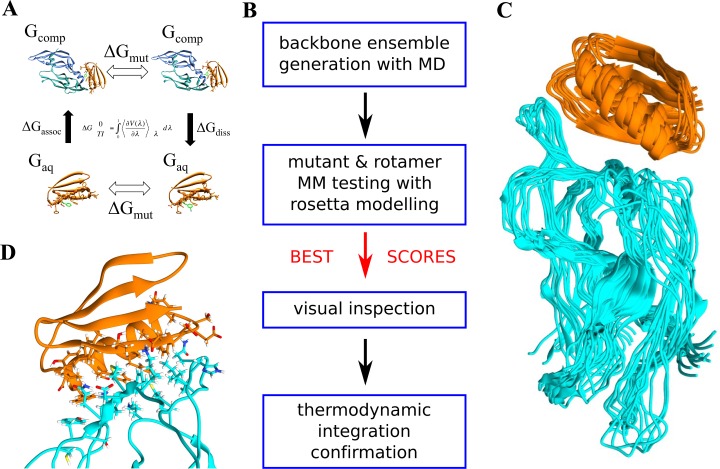
Design process for VEGF inhibiting D-protein with non-canonical amino acids. A: schematic of the four sets of calculations performed in the thermodynamic integration step. Micro-canonical ensembles are generated for both wild type and mutated structures (ΔGmut) in bound and unbound states (ΔGassoc & ΔGdiss). Output energies are integrated using the equation shown to derive ΔG. See TI methods subsection. B: the four main design steps (MM = molecular model). C: complex ensemble generated by MD followed by clustering, representing the explored conformational space; D: interface residues identified for design in atomic resolution.

Mutations with negative energy score changes that survived visual inspection were subject to TI calculations. TI computes the free energy difference between two states, in this case between the VEGF dimer in complex with the ‘native’ D-protein inhibitor and the same dimer in complex with a mutated version of the D-protein. Fig 1A shows the typical thermodynamic cycle that is used for TI. Divided into left and right are the ΔG dissociation and association processes for VEGF with the two different D-protein versions (wild-type or mutant). Horizontal processes (ΔGmut) involve the chemical changes from wild-type residue to mutant residue both for the VEGF-bound D-protein and the D-protein in the unbound state. Importantly, this step allows calculation of any stability changes that result from mutation of the apo D-protein. Destabilizing mutations result in significant conformational changes that make the TI approach unsuitable, as it is then very difficult to sample enough phase space to get converged output. For this reason, apo D-proteins with a backbone RMSD to the average that exceeded 4.0 Å were not allowed to proceed to full TI. For each ΔGmut process there are 3 main stages: 1. Partial charges are gradually switched off. 2. vdW transformation: the native residue is gradually phased out and the mutant residue simultaneously phased in 3. Partial charges are gradually switched back on again. The calculation is separated in this way because having a nonzero charge on an atom–while the vdW interactions with its surrounding are getting weaker–leads to simulation instability. For each stage, the system is first minimized and equilibrated. The equilibration step involves simulating both wild-type and mutant variants in the apo state. TI is unsuitable for measuring significant reductions in stability due to the associated increase in conformational variation. Destabilizing variants would thus be discarded, although no significant destabilization was detected, likely due to the surface location (destabilizing mutations tend to be core residues). Following equilibration, for each of stages 1–3, graduation is achieved by performing separate simulations at discrete points denoted ‘&lambda’ in the transformation. This series of predetermined windows provides the ‘coupled potential function’ and the integration is carried out over the average of the &lambda derivative of the coupled potential function at given &lambda values. TI calculations were carried out using largely the same simulation conditions described above in **Model preparation and MD simulation**. However, bonds to hydrogen were not constrained using SHAKE[32] during TI calculations, so a 1 fs time step was necessary to capture this fast motion (in the original simulation SHAKE was switched on and a 2 fs time step was allowed). Mutant transformations were simulated for both the un-complexed protein in water and in complex with VEGF. Nine windows were used for each transformation. This was carried out for each of the three main stages, namely charges switched off, phasing, charges switched back on. This means a total of 54 MD simulations are required for each full TI calculation. In the vdW transformation step simulations, softcore potentials are used, which modify the Lennard-Jones equation to prevent the origin singularity type of free energy divergence from occurring[43]. The multisander capability in Amber was used to create two groups–corresponding to the start and end states. A mixing parameter λ was used to interpolate between perturbed and unperturbed potential functions. Example scripts and input files can be found in the supplementary material.

## Results

Our method for designing D-proteins with NCAAs employs a series of steps shown in Fig 1A. First, a high-resolution crystal/NMR 3D structure (in this case VEGF-A bound to D-RFX001) is subjected to 50 ns of molecular dynamics (MD). The resulting trajectory is used to generate a backbone ensemble by conformational clustering, such as the one shown in Fig 1B. Proteins are dynamic and often explore a range of different conformations. Generating a representative ensemble of backbones therefore increases the chances for a Rosetta mutation to find conducive interface geometry. This is especially important for NCAAs because they often have unusual shapes and sizes. Each backbone is treated equally, but it should be noted that their root conformational clusters are not equally populated. Next, for each residue at the VEGF-A interface, multiple side-chain rotamers from the Dunbrack library[38] are tested and scored using Rosetta. This is repeated for all members of the backbone ensemble. For the VEGF-A inhibitor D-RFX001 (orange), 18 residues were identified as candidates for design by proximity of <5Å to any VEGF-A (cyan) residue as shown in Fig 1C. Rosetta scores for each mutated residue were compared to native scores and mutations with lower energy were visually inspected. After discarding visually poor candidates, thermodynamic integration calculations were conducted on the remaining strong mutations to confirm the energy change. Fig 1D shows a schematic of TI theory (see **Methods** for full procedure details).

**Fig 2:**
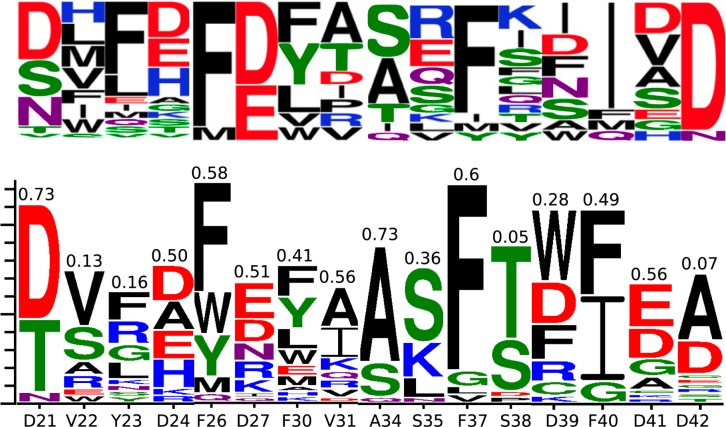
Comparison of phage display results (top) and Rosetta predictions (bottom) for optimal VEGF binding using canonical amino acids. Similarity to phage display is annotated above each logo column (Pearson correlation). Phage results are adapted from figure 2D of Mandal et al[26].

A high-resolution (1.4Å) crystal structure of VEGF-A in complex with an inhibiting D-protein was already available in the Protein Data Bank (PDB accession codes: 4GLN & 4GLS). Inhibitor binding had already been optimised with regular amino acids using mirror image phage display[26]. Before attempting to redesign the binding region using NCAAs, we first benchmarked our proposed design method using the phage display optimisation results. Logos in Fig 2 show the Rosetta predictions for the libraries previously used in phage display. Manual curation by visual inspection of the structures was used to remove Rosetta artefacts. Similarity scores (marked atop each logo column) were calculated using Pearson correlation against the phage display results. A number between 0 and 1 was assigned to every amino acid type (x 20) proportional to the height of its logo character representation. Pearson correlations between the resulting 20-value vectors for experiment and Rosetta prediction were then computed for each position. While not identical to the experimental logos, dominant themes are identified such as the preference for hydrophobic residues at positions V22, Y23, F26, F30, F37 and F40. A close comparison reveals that most of the dominant amino acids at each position match the experimental report. The computational method predicts a phenylalanine over isoleucine at residue 40, in conflict with the phage display result. However, this agrees with a point mutant affinity experiment, published in the phage display study[26]showing no F40I improvement.

TI was carried out on the manually curated Rosetta mutations with the largest negative energy change. Table 1 lists results of the TI calculations. Of particular note is mutation Y23F, which was also previously subject to additional point mutant affinity experiments, and showed binding energy change commensurate with the TI result. Also notable are results comparing D21 with T21, V22 with S22, F23 with R23, F26 with W26, D27 with E27, F30 with Y30, I31 with A31, S38 with T38, F40 with I40, and D42 with A42. In each case the preferred residue matches the preferred residue from phage display. Both phage display and Rosetta + TI also confirm negative charge conservation at residues 27 and 42. Together, this suggests that Rosetta scoring across backbone ensembles–combined with visual inspection and TI–allows the recapitulation of experimental outcomes.

**Table 1:** Results of thermodynamic integration calculations to test Rosetta predictions. Left row lists the initial residue starting points. Top row header shows the mutation residue identity. Binding energy change units are kcal/mol.

	D	E	T	K	F	R	I	S	A	V	Y	W
Library 1												
**D21**	N		+0.3									
**V22**								+0.3		N		
**Y23**					-1.5	-0.4						
**D24**	N	+0.2							+0.8			
Library 2												
**F26**					N							+0.7
**D27**	N	+0.5										
**F30**					N						+0.0	
**V31**							-0.8		-0.2			
Library 3												
**A34**								-0.3	N			
**S35**				-0.2				N				
**F37**					N							
**S38**			+0.7					N				
Library 4												
**D39**	N				-0.1							-0.6
**F40**					N		-0.2					
**D41**	N	-0.0										
**D42**	N								+1.1			

**Fig 3:**
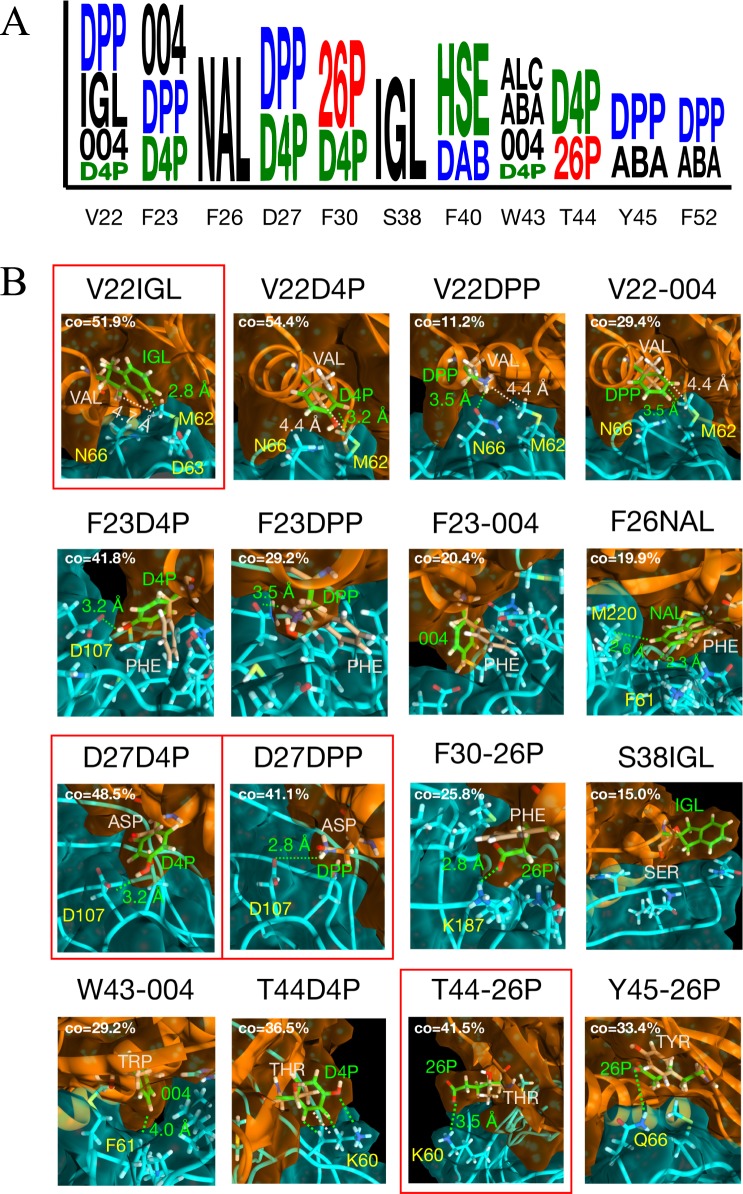
Rosetta energy and structure predictions for VEGF binding using non-canonical amino acids. A: logo showing the NCAA mutations that produce a lower energy score than the lowest energy CAA at each position. Character size is proportional to the magnitude of change in Rosetta energy score. B: interaction changes yielding improvements for each NCAA that has lower binding energy score than the best CAA. Inhibitor is shown in orange, VEGF is shown in cyan. Original CAAs are coloured beige and NCAA mutations are lime green. Red boxes highlight the most significant energy changes, confirmed by subsequent TI. The dominant conformational state from each simulation is shown (from clustering at the interface). Each dominant cluster occupancy time (CO) is shown as the percentage of total simulation time.

With the methodology successfully benchmarked using experimental results, we next sought to introduce NCAAs using the same approach. We identified a more broadly defined interface than the benchmarking calculations, reasoning that larger NCAAs may be capable of accessing VEGF surface topology that is inaccessible to CAAs. To this end the interface was defined as any D-RFX001 residue <5Å from the VEGF surface. This was calculated for all members of the MD generated ensemble as well as the crystal structure. In total, 18 residues were identified for design (Fig 1C). Each position was mutated to each of 114 NCAAs and scored using Rosetta. As with the CAAs, multiple rotamers were tested for each NCAA at each design position. Structures from Rosetta with improved energy scores were again manually curated by visual inspection. This step was carried out according to criteria described in **Methods** and led to 59% of the candidates from Rosetta being removed. The logo in Fig 3A shows the remaining NCAAs with a lower binding energy score than the lowest energy CAA for that position. Of the 18 positions tested, an NCAA was predicted to improve 11 positions (only these are shown in the logo). The positions are: 22, 23, 26, 27, 30, 38, 40, 43, 44, 45, and 52. Fig 3B shows a selection of structure ‘close-ups’ showing the improved interaction of NCAA over CAA. The most successful NCAA mutations were predominantly large hydrophobic groups such as IGL, D4P, 004 and NAL, or charged groups–both negative (26P) and positive (DPP). Mutation of Val at position 22 to the large hydrophobic IGL facilitates more extensive burying of the hydrophobic M11 and Y14 residues on VEGF. A similar story emerges for mutation to 004 at the same position, whereby this shortened version of Phe is able to fit more snugly with the VEGF surface than either native Val or regular Phe. D4P (a shortened version of Tyr) substitutions at 23 and 27 are more complicated as the hydroxyl is involved in polar interactions in addition to a hydrophobic patch. Broadening the interaction area did yield additional contact as mutation to the negatively charged 26P at new position 44 allowed salt bridge formation with K9 on VEGF. Other electrostatic interactions were created by NCAA substitution at positions 27 by DPP, and 30 by 26P.

Each of the lower energy score candidates that survived manual curation were further tested using TI. The results of 26 TI mutations measured are shown in Table 2. Full names and 2D structures for each of the 10 NCAA types that show improved binding energy are shown in S1 Table in the supporting information. Of the 26 mutations tested, 16 predictions were confirmed by TI to improve binding. 8 of the 10 mutations not confirmed by TI were hydrophobic-to-hydrophilic, compared to only 2 of the 16 confirmed. This indicates a possible systematic bias in the Rosetta predictions with respect to NCAAs, likely a consequence of the lack of good solvent accountability in the calculation.

**Table 2:** Results of thermodynamic integration calculations to test Rosetta predictions for better binding non-canonical mutations. Left row lists the initial residue starting points. Top row header shows the non-canonical residue mutation identity. Binding energy change units are kcal/mol. The most significant energy changes are underlined.

	DPP	IGL	004	D4P	NAL	26P	HSE	DAB	ALC	ABA
**V22**	-1.24	-2.84	-0.51	-1.20						
**F23**	-0.61		+1.21	-1.12						
**F26**					-1.6					
**D27**	-4.25			-3.72						
**F30**				+1.01		+2.18				
**S38**		-0.35								
**F40**							+2.81	+3.22		
**W43**			-1.05	-1.01					-0.89	+0.35
**T44**				-1.15		-3.70				
**Y45**	+0.97					-0.49				+1.11
**F52**	+1.37									+0.50

Following the round of individual point mutations, combinations of the lowest energy mutations from TI were calculated using a further round of TI. Lowest energy mutations were those with a negative energy change of >2.0 kcal/mol and four combinations met this criterion (underlined in Table 2). Table 3 shows the outcome for each combination.

**Table 3:** Results of thermodynamic integration calculations on double and triple mutant. Left row lists the initial residue starting points. Binding energy change units are kcal/mol.

**Double mutants**														
	**Combo 1**			**Combo 2**			**Combo 3**				**Combo 4**		**Combo 5**	
**V22**	IGL	-		IGL	-		IGL		-		-	-	-	-
**D27**	DPP	-		-	D4P		-		-		DPP	-	-	D4P
**T44**	-	-		-	-		-		26P		-	26P	-	26P
	-4.42			-3.41			-6.68				-6.74		-6.25	
**Triple mutants**														
	**Combo 1**					**Combo 2**								
**V22**	IGL		-	-		IGL		-		-				
**D27**	-		DPP	-		-		D4P		-				
**T44**	-		-	26P		-		-		26P				
	-6.97					-7.18								

**Fig 4:**
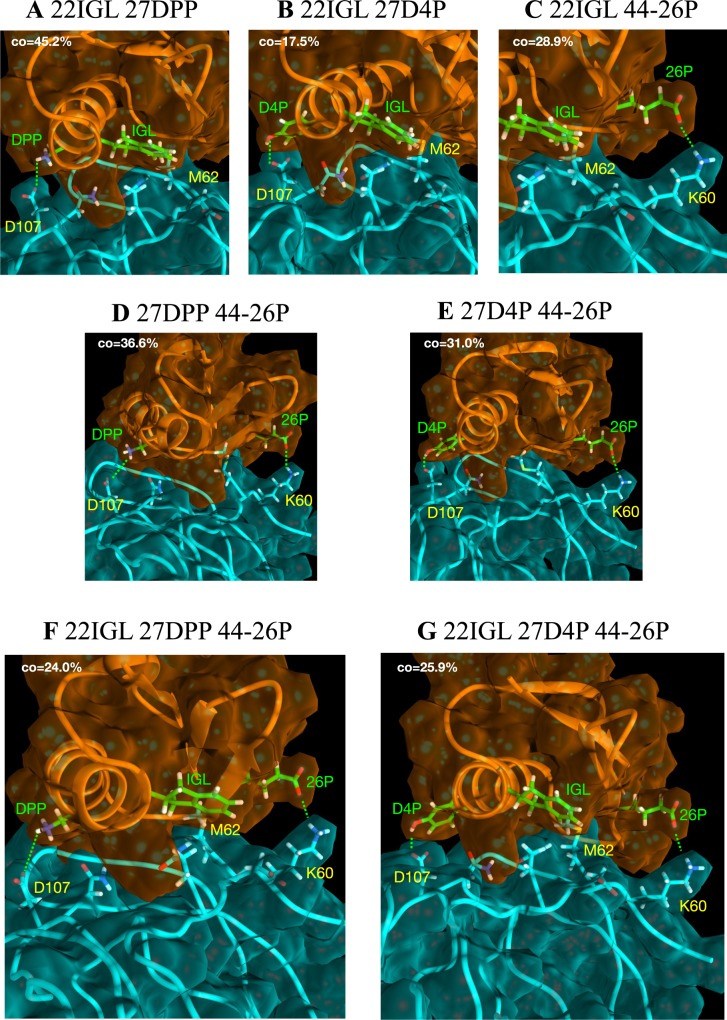
Non-canonical amino acid double and triple mutant structures produces by Rosetta. A-E: double mutants. F-G: triple mutants. Inhibitor is shown in orange, VEGF is shown in cyan. NCAAs shown in lime green. The dominant conformational state from each simulation is shown (from clustering at the interface). Each dominant cluster occupancy time (CO) is shown as the percentage of total simulation time.

It is interesting that for double mutants in 4 of 5 cases the improvement in binding affinity was less than the sum of individual point mutation changes. Combination one involves both increased hydrophobic patch facilitation by V22IGL, and bifurcated hydrogen bond/salt bridge formation by D27DPP (Fig 4A). While the combination does predict improved affinity, the effect is not entirely additive. This is because the optimal interaction distance and orientation for either mutated residue renders the other suboptimal–by subtle changes in the global D-RFX001–VEGF binding orientation. Combination 2, combining mutations V22IGL and D27D4P, actually reduces the binding energy change to less than that of the single D27D4P mutation (-3.41 and -3.72 kcal/mol respectively). This appears to be a consequence of V22IGL effecting an orientation change that precludes hydrogen bond formation by the D4P hydroxyl group with a VEGF aspartic acid (Fig 4B). Combination 3 also involves V22IGL. However, orientation change is tolerated by 26P and its interacting VEGF lysine due to the flexibility of these groups. They are able to adopt alternative rotamers such that a salt bridge still forms (Fig 4C). Accordingly, the combined energy change slightly exceeds that of the single mutants summed, at -6.68 kcal/mol. Similarly, combinations 4 and 5 also involve this flexible pair and the lack of contingency between the mutant residues is reflected in binding energy changes that more closely approaches the sum of the single mutant changes; -6.74 and -6.25 kcal/mol respectively (Fig 4D and 4E).

Using the same four mutations, both possible triple mutants were next modelled and evaluated with TI. Triple mutant 1 suffers the same orientation issues as double mutant 1 with respect to V22IGL and D27DPP (Fig 4F). However, the 26P interaction is less influenced, resulting in a combined interaction energy change of -6.97 kcal/mol. Triple mutant 2 is only marginally better at -7.18 kcal/mol as the effect of IGL on D4P, especially when combined with 26P, means D4P is largely precluded from contributing to the interaction (Fig 4G).

## Discussion

We suggest an *in silico* design approach to the problem of accurately improving the binding affinity of D-protein based inhibitors with NCAAs. By sequentially performing Rosetta modelling, visual inspection, and TI MD (eliminating the poor candidates at each step) we identify D-NCAAs that optimize affinity. This approach is not limited by target size and can be extended to new NCAAs as they become available. Interestingly, a small group of 5 NCAAs emerge as improving binding at multiple positions, namely DPP, IGL, 004, D4P and 26P. These residues are a pleasing mix of negatively charged, positively charged, hydrophobic and amphiphilic species. An examination of the structures (S1 Table) reveals that they are strikingly similar to CAAs and this may partially explain their facile integration. It should be noted that the visual inspection is time consuming and potentially subject to human error. Automating this step could therefore be beneficial. This could be attempted in the future by constructing a more sophisticated scoring method, such as training a neural network on known good and bad arrangements of atoms at interfaces.

DPP is a severely shortened version of Lys with the four-carbon chain reduced to only one. This facilitates electrostatic and polar interaction that would be precluded with the three canonical positively charged residues by virtue of it’s reduced bulk. It also offers different options with respect to optimal interaction angles. The chain reduction also removes the hydrophobic section of the side chain extant for both Arg and Lys, which imparts a degree of advantage in highly polar environments. These features are exemplified by the D27DPP mutation that replaces a negatively charged Asp that at best forms a suboptimal hydrogen bond with Y65. The replacement DPP forms a bifurcated interaction involving both a hydrogen bond with N106 and salt bridge formation with D107. This mutation yields the best improvement in binding affinity according to TI calculations (Table 2). DPP substitution of V22 introduces a hydrogen bond to Q66 analogous to the predicted canonical Ser interaction (Fig 2). While very similar, DPP performs better than Ser. This is counterintuitive as R-OH–O hydrogen bonds are stronger than R-NH–O although the result aligns with phage display results, where a His performs best at this position (Fig 2). The other DPP substitutions suggested by Rosetta (at 23, 45 & 52) are less convincing and this is reflected in the TI results.

IGL is similar to Trp, primarily differentiated by the absence of a heterocyclic nitrogen, saturated pentameric ring, and ring attachment to Cα rather than Cβ. This shortening slightly reduces the bulk of IGL and allows subtly different peptide topology. This explains the significant improvement of V22IGL where the patch with M62 would be a poorer surface with the extra Trp bulk. The 004 NCAA is a shortened version of Phe with the ring attached to the Cβ. This again presents subtle topological differences and for example facilitates much improved surface complementarity over Trp at residue 43. A substitution at residue 23 with the same NCAA also improves fit over Phe but reduces proximity to the local hydrophobic elements of VEGF surface (Y65 & Y69). This is reflected in unfavorable TI results and highlights a possible limitation of the Rosetta scoring function.

Following the emerging theme of size reduction, D4P is a shortened version of Tyr and therefore behaves very similarly to 004. However, the hydroxyl group also allows distances and angles for hydrogen bond formation–subtly different to any CAA (F23D4P, D27D4P are good examples). The 26P NCAA is similar to Arg in size but carries a negative rather than positive charge by virtue of three carboxyls instead of a guanidinium group. This allows a much greater reach for salt bridges and hydrogen bonds than Asp or Glu. This is demonstrated by the T4426P substitution, which extends the binding interface by forming a strong new electrostatic interaction with K60. These observations suggest that one approach to PPI optimization might be the testing of different length versions of each interacting residue. This is supported by a study of HIV-1 Tat-derived peptides, which showed that varying Arg methylene chain length could both diminish and enhance binding specificity and cell penetration [44]. Our work predicts this approach could be usefully extended to other side chain types.

Using an ensemble of backbone structures representative of the protein’s full conformational space means the number of beneficial mutations predicted by Rosetta is high, with many false positives. The Rosetta scoring function does not handle solvent effects very well, which is one reason for this. By using TI as a filter–with explicit solvent–this number can be reduced to a set that is more tractable from an experimental validation perspective. In the experience of the authors, less precise filters (e.g. FoldX[45]) generally perform little better that the Rosetta energy scoring function. Our protocol could likely be improved in the future by clustering from simulations that entail a beneficial mutation. An NCAA often influences the interface such that new backbone conformational space is explored. Any new ensemble may therefore allow different adjacent mutations–not possible in the initial round. Iterative recycling of beneficial mutations over many rounds–using both CAAs and NCAAs–could be even more effective in optimizing the interface.

## Conclusions

Incorporation of D-amino acids (especially D-NCAAs) is precluded for the majority of drug targets using current experimental protein design methods. The computational approach presented here allows access to inhibitor design using D-NCAAs for cases where the target binding domain structure has been solved. Using the available structure of a D-protein inhibitor D-RFX001 in complex with the VEGF homodimer, we identified D-NCAAs that are predicted to improve binding affinity by as much -7.18 kcal/mol. Further work is necessary to experimentally validate these predictions, which, if born out, will equate approximately 10^5^ -fold improvement in Kd with the important VEGF target.

## Supporting information

S1 Table Chemical names and 2D structures for each of the 10 NCAAs that improve binding energy. (PDF)
