# Supplementary material for: A computational approach for designing D-proteins with non-canonical amino acid optimised binding affinity
Source: PLoS One. 2017 Nov 6;12(11):e0187524. doi: 10.1371/journal.pone.0187524 (PMC5673230; doi:10.1371/journal.pone.0187524)
Supplement: S1 Table — (PDF) [file pone.0187524.s001.pdf]

NCAA name	Code	Structure
2,3-diaminopropanoic acid	DPP	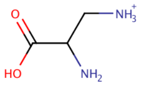
2-indanyl-glycine	IGL	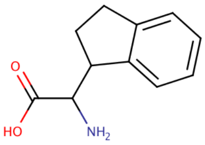
Phenylglycine	004	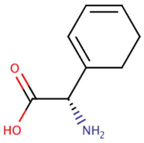
4-hydroxyphenylglycine	D4P	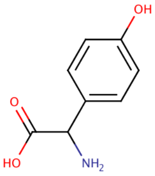
2-napthylalanine	NAL	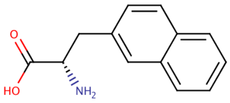
2-amino-6-oxopimelic acid	26P	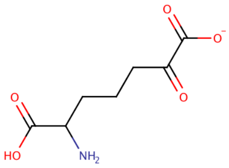
Homoserine	HSE	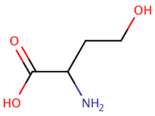
Diaminobutyric acid	DAB	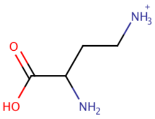
Cyclohexylalanine	ALC	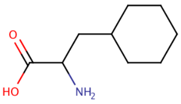
2-aminobutyric acid	ABA	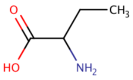
